# Supplementary material for: Leucettinib-21 decreases dosage effects of DYRK1A in human trisomy 21 iPSC-derived neural cells
Source: bioRxiv. 2026 Feb 5:2026.02.05.704014. Preprint. [Version 1] doi: 10.64898/2026.02.05.704014 (PMC12889579; doi:10.64898/2026.02.05.704014)
Supplement: 1 [file NIHPP2026.02.05.704014v1-supplement-1.pdf]

bioRxiv preprint doi: https://doi.org/10.64898/2026.02.05.704014; this version posted February 5, 2026. The copyright holder for this preprint (which was not certified by peer review) is the author/funder, who has granted bioRxiv a license to display the preprint in perpetuity. It is made available under aCC-BY 4.0 International license.

Supporting Tables

Supporting Table 1. Media Recipes and Components

Human Embryonic Stem Cell (hESC) Media			
**Component**	**Manufacturer**	**Catalog #**	**Volume/Concentration**
DMEM/F12	Gibco	11330	400 mL
KOSR	Gibco	10828	100 mL
MEM NEAA (100x)	Gibco	11140-050	5 mL
L-Glutamine (200 mM)	Gibco	25030-061	2.5 mL<br>1 mM final
FGF-2	Waisman Biomanufacturing	-	20 µL<br>4 ng/mL final
2-Mercaptoethanol	Millipore Sigma	M6250	7 uL (in 5 mL L-Glutamine)

Neural Progenitor Cell (NPC) Media			
**Component**	**Manufacturer**	**Catalog #**	**Volume/Concentration**
Neurobasal	Gibco	21103049	400 mL
N-2 Supplement	UW Human Stem Cell Core	-	5 mL
B-27 Supplement (50x)	Gibco	12587010	5 mL
L-Glutamine (200 mM)	Gibco	25030-061	5 mL
Antibiotic-Antimycotic (100x)	Gibco	15240-062	5 mL

Neural Induction Media (NIM)			
**Component**	**Manufacturer**	**Catalog #**	**Volume/Concentration**
DMEM/F12	Gibco	11330	240 mL
Neurobasal	Gibco	21103049	240 mL
N-2 Supplement	UW Human Stem Cell Core	-	5 mL
L-Glutamine (200 mM)	Gibco	25030-061	5 mL
Antibiotic-Antimycotic (100x)	Gibco	15240-062	5mL
SB 431542	Biogems	3014193	5 µL<br>10 µM final
XAV 939	Tocris	3748	5 µL<br>0.1 µM final
LDN-193189 2HCl	Selleckchem	S7507	5 µL<br>2 µM final

bioRxiv preprint doi: https://doi.org/10.64898/2026.02.05.704014; this version posted February 5, 2026. The copyright holder for this preprint (which was not certified by peer review) is the author/funder, who has granted bioRxiv a license to display the preprint in perpetuity. It is made available under aCC-BY 4.0 International license.

**Neural Differentiation Media (NDM)**			
**Component**	**Manufacturer**	**Catalog #**	**Volume/Concentration**
Neurobasal	Gibco	21103049	480 mL
N-2 Supplement	UW Human Stem Cell Core	-	5 mL
B-27 Supplement (50x)	Gibco	12587010	5 mL
Antibiotic-Antimycotic (100x)	Gibco	15240-062	5 mL
GlutaMax Supplement (200mM)	Gibco	35050061	5 mL
30% Glucose (dissolved in Neurobasal)	Dot Scientific	DSG32040-500	5 mL
BDNF	Peprotech	450-02	50 µL<br>10 ng/mL final
GDNF	Peprotech	450-10	50 µL<br>10 ng/mL final
L-Ascorbic Acid	Millipore Sigma	A0278	500 uL<br>200 ng/mL final
Cyclic AMP	Millipore Sigma	D0260	500 uL<br>1 µM final

Supporting Table 2. Immunocytochemistry and Western Blot Antibodies

**Immunocytochemistry Antibodies**			
**Antibody**	**Manufacturer**	**Catalog #**	**Dilution**
Ms α DYRK1A	Abnova	H00001859-M01	1:500
Rb α PAX6	BioLegend	901301	1:500
Ms α SOX2	R&D Systems	MAB2018	1:500
Rb α NEUN	Abcam	ab104225	1:1000
Ch α MAP2	Abcam	ab5392	1:5000
Rb α pT286-Cyclin D1	Cell Signaling Technology	3300S	1:500
Rb α Ki-67	Cell Signaling Technology	9129	1:400
Rb α Tau (phospho T212)	Abcam	ab4842	1:500
Ms α Tau (HT7)	Invitrogen	MN1000	1:500
Rb α TBR1 [EPR8138(2)]	Abcam	Ab183032	1:500
Dn α Ms 488	Invitrogen	A11055	1:500
Dn α Ms 488	Invitrogen	A21202	1:500
Dn α Ms 488	Invitrogen	R37114	1:500
Dn α Rb 546	Invitrogen	A10040	1:500
Dn α Ch 647	Invitrogen	A78952	1:500
Gt α Ms 488	Invitrogen	A11029	1:500
Gt α Rb 546	Invitrogen	A11035	1:500

bioRxiv preprint doi: https://doi.org/10.64898/2026.02.05.704014; this version posted February 5, 2026. The copyright holder for this preprint (which was not certified by peer review) is the author/funder, who has granted bioRxiv a license to display the preprint in perpetuity. It is made available under aCC-BY 4.0 International license.

**Western Blot Antibodies**			
**Antibody**	**Manufacturer**	**Catalog #**	**Dilution**
Ms α DYRK1A	Sigma-Aldrich	WH00001859M1 (clone 7D10)	1µg / mL in 5% BSA (overnight 4°C)
Ms α GAPDH	ThermoFisher	AM4300	1/30,000 in 5% BSA (2h RT)
Rb α pT286-Cyclin D1	Cell Signaling Technology	3300S	1/2,000 in 5% BSA (overnight 4°C)
Rb α Cyclin D1	Cell Signaling Technology	2922	1/2,000 in 5% BSA (overnight 4°C)
Ms α Tau (HT7)	Innogenetics	90222	1/1,000 in 5% BSA (2h RT)
Ms α GSK3 α/β	Enzo Life Sciences	ADI-KAM-ST002 (clone 1H8)	1/2,000 in 5% BSA (overnight 4°C)
Rb α Phospho-Tau (Thr212)	Fisher Scientific	44-740G	1/2,000 in 5% BSA (overnight 4°C)
Rb α Tau (phospho Thr217)	Abcam	ab192665	1/2,000 in 5% BSA (overnight 4°C)
Gt α Rb IgG (H+L) HRP Conjugate	Bio-Rad	1706515	1/4,000 (1h RT)
Gt α Ms IgG (H+L) HRP Conjugate	Bio-Rad	1706516	1/4,000 (1h RT)

bioRxiv preprint doi: https://doi.org/10.64898/2026.02.05.704014; this version posted February 5, 2026. The copyright holder for this preprint (which was not certified by peer review) is the author/funder, who has granted bioRxiv a license to display the preprint in perpetuity. It is made available under aCC-BY 4.0 International license.

Suporting Figure 1

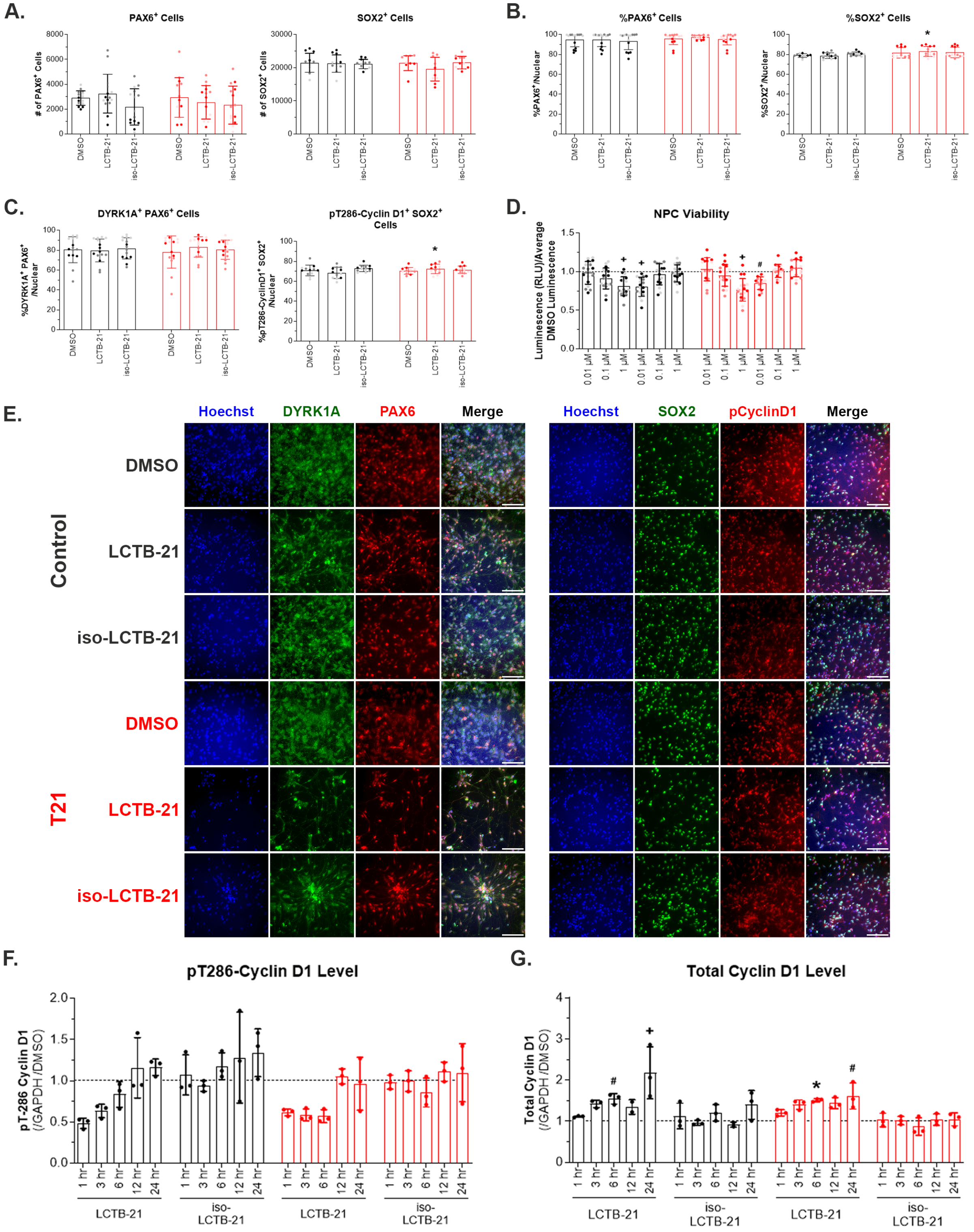

bioRxiv preprint doi: https://doi.org/10.64898/2026.02.05.704014; this version posted February 5, 2026. The copyright holder for this preprint (which was not certified by peer review) is the author/funder, who has granted bioRxiv a license to display the preprint in perpetuity. It is made available under aCC-BY 4.0 International license.

Supporting Figure 1.

A) Pax6+ and Sox2+ progenitor cells in culture. Treatment with LCTB-21 has no effect on the number of NPCs in culture. B) Percent of Pax6+ and Sox2+ cells in culture. Treatment with LCTB-21 has no effect on the percentage of NPCs in culture relative to respective DMSO. There is a slight increase in LCTB-21 treated T21 NPCs compared to LCTB-21 treated control NPCs. C) Percent of DYRK1A expressing Pax6+ cells and pT286-cyclin D1 expressing Sox2+ cells. Treatment with LCTB-21 has no effect on the percentage of DYRK1A expressing or pT286-cyclin D1 expressing NPCs in culture. There is a slight increase in pT286-cyclin D1+Sox2+ T21 NPCs treated with LCTB-21 compared to LCTB-21 treated control NPCs. D) NPC viability. There is a slight decrease in the viability of NPCs treated with 1 μM LCTB-21 and 0.01 μM iso-LCTB-21 relative to respective DMSO (represented by the dashed line). E) Representative images of DYRK1A+ Pax6+ NPCs and pT286-cyclin D1+ Sox2+ NPCs (20x objective, 100 μm scale bar). F) pT286-cyclin D1 levels over time. pT286-cyclin D1 is reduced in control and T21 NPCs within 1 hour of treatment with LCTB-21 and this reduction persists for 6 hours compared to respective DMSO treatment (represented by the dashed line). G) Total cyclin D1 levels over time. Total cyclin D1 increases in control and T21 NPCs within 6 hours after treatment with LCTB-21 compared to respective DMSO treatment (represented by the dashed line). Data presented as mean + SD. P-values from Two-way ANOVA with post-hoc tests are presented as * $\leq$ 0.05, # $\leq$ 0.01, X $\leq$ 0.001, + $\leq$ 0.0001. Two-way ANOVA results, post-hoc tests, and P-values, and excluded values are listed in Supporting Table 3.

bioRxiv preprint doi: https://doi.org/10.64898/2026.02.05.704014; this version posted February 5, 2026. The copyright holder for this preprint (which was not certified by peer review) is the author/funder, who has granted bioRxiv a license to display the preprint in perpetuity. It is made available under aCC-BY 4.0 International license.

Supporting Figure 2

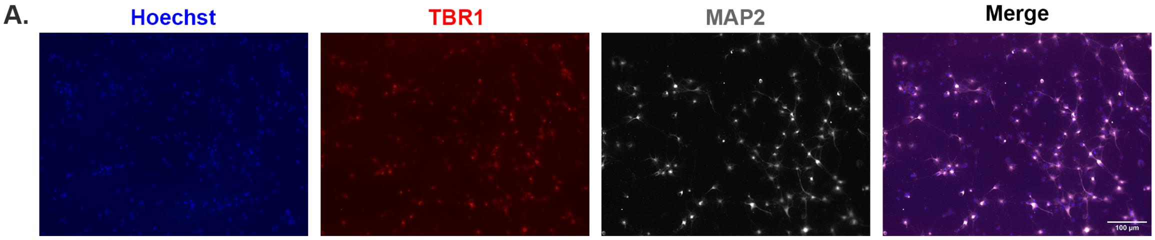

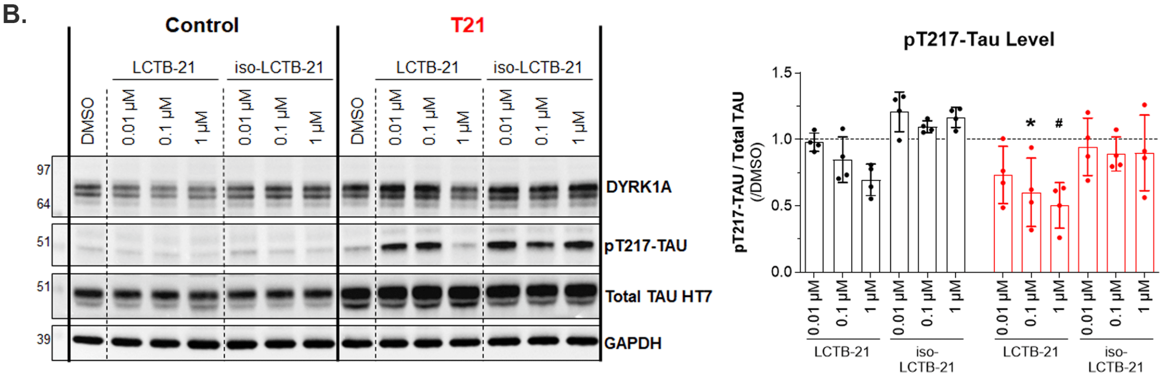

bioRxiv preprint doi: https://doi.org/10.64898/2026.02.05.704014; this version posted February 5, 2026. The copyright holder for this preprint (which was not certified by peer review) is the author/funder, who has granted bioRxiv a license to display the preprint in perpetuity. It is made available under aCC-BY 4.0 International license.

Supporting Figure 2.

A) TBR1 expression in neurons, confirming the differentiation of iPSCs into cortical neurons (20x objective, 100 μm scale bar). B) Western blot and quantification of Total Tau and pT217 Tau levels in control and T21 cortical neurons. pT217 Tau levels decrease as DYRK1A activity decreases with increasing doses of LCTB-21. Data presented as mean + SD. P-values from Two-way ANOVA with post-hoc tests are presented as * $\leq$ 0.05, # $\leq$ 0.01, X $\leq$ 0.001, + $\leq$ 0.0001. Two-way ANOVA results, post-hoc tests, and P-values, and excluded values are listed in Supporting Table 3.
